# Functionalizable Glyconanoparticles for a Versatile Redox Platform

**DOI:** 10.3390/nano11051162

**Published:** 2021-04-29

**Authors:** Marie Carrière, Paulo Henrique M. Buzzetti, Karine Gorgy, Muhammad Mumtaz, Christophe Travelet, Redouane Borsali, Serge Cosnier

**Affiliations:** 1 UMR 5250, Département de Chimie Moléculaire, CNRS, Université Grenoble Alpes, CEDEX 09, 38058 Grenoble, France; marie.carriere@univ-grenoble-alpes.fr (M.C.); paulo-henrique.maciel-buzzetti@univ-grenoble-alpes.fr (P.H.M.B.); karine.gorgy@univ-grenoble-alpes.fr (K.G.); 2 CERMAV, UPR 5301, CNRS, Université Grenoble Alpes, CEDEX 09, 38058 Grenoble, France; mumtaz@cermav.cnrs.fr (M.M.); christophe.travelet@cermav.cnrs.fr (C.T.)

**Keywords:** glyconanoparticles, block copolymer, β-cyclodextrin, maltoheptaose, host–guest interaction, anthraquinone sulfonate

## Abstract

A series of new glyconanoparticles (GNPs) was obtained by self-assembly by direct nanoprecipitation of a mixture of two carbohydrate amphiphilic copolymers consisting of polystyrene-block-β-cyclodextrin and polystyrene-block-maltoheptaose with different mass ratios, respectively 0–100, 10–90, 50–50 and 0–100%. Characterizations for all these GNPs were achieved using dynamic light scattering, scanning and transmission electron microscopy techniques, highlighting their spherical morphology and their nanometric size (diameter range 20–40 nm). In addition, by using the inclusion properties of cyclodextrin, these glyconanoparticles were successfully post-functionalized using a water-soluble redox compound, such as anthraquinone sulfonate (AQS) and characterized by cyclic voltammetry. The resulting glyconanoparticles exhibit the classical electroactivity of free AQS in solution. The amount of AQS immobilized by host–guest interactions is proportional to the percentage of polystyrene-block-β-cyclodextrin entering into the composition of GNPs. The modulation of the surface density of the β-cyclodextrin at the shell of the GNPs may constitute an attractive way for the elaboration of different electroactive GNPs and even GNPs modified by biotinylated proteins.

## 1. Introduction

Amphiphilic block copolymers (BCPs) are ideal building blocks for materials science. Their self-assembly has attracted great attention for many years because they can provide in solution different three-dimensional morphologies such as spherical micelles, vesicles, cylindrical micelles, lamellae and others structures that have attractive applications in biomaterials, catalysis, photoelectronic materials, biomedicine and more recently, in bioelectrochemistry applications [1,2,3,4]. To obtain nanoparticles with diblock copolymers, which are composed of two chemically hydrophobic and hydrophilic different blocks, the copolymers were dissolved in a first step in a mixture of solvents, allowing the solubilization of the different blocks. The next step consists of adding the resulting solution to a large volume of water, followed by organic solvent removal by evaporation [4] or by dialysis [3], leading to self-assembled NPs. Depending on the targeted application, the introduction of a specific group can generate nanoparticles with specific properties. For example, the linkage of a polystyrene chain with a cyclodextrin, leading to a polystyrene-block-β-cyclodextrin copolymer as already described, allowed the formation of micelles by nanoprecipitation in water with a polystyrene core covered by a shell formed by cyclodextrins. In addition, if a hydrophobic compound is added in the organic phase containing the copolymer, it can be incorporated in the core of the nanoparticles as well as inserted in the cyclodextrins (βCD) cavities by host–guest interaction, as previously reported for bis-pyrene-2,2′-Azino-bis(3-ethylbenzothiazoline-6-sulfonic acid) [3], 9,10-phenanthrenequinone (PQ) [4], or tetrazines (TZ) [5].

Another approach consists of the post-functionalization of these glyconanoparticles in an aqueous medium with molecules that are soluble or partially soluble in water. In this way, for bioelectrochemical applications, the nanoparticles can constitute a versatile platform that may be easily functionalized by compounds capable of orienting or electrically wiring enzymes. In addition, this concept can be extended to biological macromolecules sensitive to organic solvents such as enzymes, antibodies or oligonucleotides. Their functionalization by molecules offering binding affinities with βCD will lead to the formation of nanoparticles modified by a shell of biomolecules. Although metallic nanoparticles coated with cyclodextrin have been modified by enzymes such as adamantane modified L-phenylalanine dehydrogenase or bovine pancreatic trypsin [6,7], no modification of polystyrene-block-β-cyclodextrin-based nanoparticles by proteins has been described.

With this objective, an interesting parameter may be the surface density of the inclusion site at the surface of the nanoparticle shell. Replacing the βCD with a linear saccharide should allow a reduction in the number of associations with proteins which are large size molecules and therefore limit alterations in their flexibility and their activity. Moreover, compared to the toroidal form of β-cyclodextrin, a linear saccharide could form a more regular layer and thus facilitate the formation of a more compact hydrophilic shell for the nanoparticles.

In this context, and in order to modulate the amount of βCD at the surface of glyconanoparticles (GNPs), we report a series of new GNPs obtained by the self-assembly of two amphiphilic glycopolymers in different proportions, namely polystyrene-block-maltoheptaose copolymer (PS-*b*-MH) [8] and polystyrene-block-β-cyclodextrin copolymer (PS-*b*-βCD). PS-*b*-MH has a linear conformation that does not display specific association such as host–guest interactions. By varying the ratios, 100% PS-*b*-βCD, 50% PS-*b*-βCD—50% PS-*b*-MH, 10% PS-*b*-βCD—90% PS-*b*-MH, it is therefore possible to generate a series of GNPs with different surface densities of βCD groups. The modulation of the surface density of βCD at GNPs and its impact on host–guest properties was investigated by incubation with anthraquinone sulfonate (AQS), a water-soluble model compound. This redox compound forms a stable host–guest complex with βCD with an association constant of 600 L mol^−1^ and can be easily detected by cyclic voltammetry [9].

## 2. Materials and Methods

### 2.1. Materials

*p*-toluenesulfonyl chloride (*regentPlus*, ≥99%), NaN_3_ (*regentPlus*, ≥99.5%), trimethylamine (TEA, 99%), 1,4-dioxane (anhydrous, 99.8%), and calcium hydride (CaH_2_, 95%), magnesium sulfate (anhydrous, *reagentPlus*, ≥99.5%), ethylene oxide solution (2.5–3.3 mol L^−1^ in THF), di-*n*-butylmagnesium solution (1.0 mol L^−1^ in heptane) and *sec*-butyllithium solution (1.4 mol L^−1^ in cyclohexane) were purchased from Sigma Aldrich (Munich, Germany)and used as received. Toluene (Biosolve, Dieuze, France) was first distilled over CaH_2_ and then over polystyryllithium. Styrene (Sigma Aldrich, *ReagentPlus,* ≥99%) was first distilled over CaH_2_ and then over di-n-dibutylmagnesium. CH_2_Cl_2_ (CP) stabilized by amylene and pyridine (extra dry) was obtained from Biosolve and distilled over CaH_2_ at 35 °C prior to use. Maltoheptaose (MH) (Hayashibara Company, Okayama, Japan) and 6-mono-o-(p-toluenesulfonyl)-β-cyclodextrin (Cyclolab, Budapest, Hungary) were functionalized with an alkynyl group according to the literature methods [10,11]. The Cu/CuO nanopowder (CuNP, 20–50 nm, 99.9% metal basis) was purchased from Alfa Aesar (Ward Hill, MA, USA). Tetrahydrofuran (THF), absolute methanol and absolute ethanol were bought from Biosolve. *N*,*N*-dimethylformamide (DMF, Fisher Scientific, Waltham, MA, USA) were used as received. Milli-Q water was obtained by water purification to a resistivity of 18.2 MΩ cm using a Millipore Ultrapure system (MilliporeSigma, Burlington, MA, USA). The deuterated solvents were purchased from Eurisotop (Saint-Aubin, France). The cuprisorb resin was from Seachem (Madison, GA, USA). Sodium anthraquinone-2-sulfonate (AQS, ≥98%) and potassium chloride (KCl, ≥99%) were purchased from Sigma Aldrich.

### 2.2. Block Copolymers Synthesis (BCPs) Protocols

PS-*b*-MH and PS-*b*-βCD block copolymers synthesis is preceded by hydroxyl-terminated polystyrene (PS-OH), tosyl terminated polystyrene (PS-OTs) and azido-functionalized polystyrene (PS-N_3_) preparation. The synthesis descriptions are described, and the characterization of the different copolymers can be found in the Supplementary Information.

#### 2.2.1. Synthesis of Hydroxyl-Terminated Polystyrene (PS-OH)

Hydroxyl-terminated polystyrene was prepared by anionic polymerization of styrene accompanied by termination with ethylene oxide. Toluene (300 mL) was introduced in a 1 L flamed dried round bottom two necked flask equipped with a magnetic stirrer and specially designed joint with roto-flow, under vacuum. Styrene (40 g, 44.15 mL) was then added, and the flask filled with argon. sec-Butyllithium (~1.4 mol L^−1^ in cyclohexane, 10 mmol, 7.14 mL) was then introduced in order to initiate the polymerization. The color of the reaction mixture turned red. The reaction flask was placed in an oil bath preheated at 35 °C for 3 h. The polymerization reaction was finally terminated by the addition of ethylene oxide (5.0 mL, ~3 mol L^−1^ solution in THF) in the reaction mixture accompanied with the addition of an excess of degassed methanol. The solvent was removed under vacuum using a rotary evaporator at 40 °C. The polymer was redissolved in an appropriate amount of THF and precipitated twice in methanol (1 L). The white precipitate of hydroxyl terminated polystyrene was filtered using a sintered glass funnel under vacuum and dried in a vacuum oven at 40 °C overnight. There was 39 g of solid product, with a 95% yield. The sample was characterized using ^1^H NMR and SEC. *M*_n_ (^1^H NMR) ~4500 g/mol, *M*_n_ (SEC, DMF) = 3800 g/mol (Figure S1).

#### 2.2.2. Synthesis of Azido-Functionalized Polystyrene (PS-N_3_)

The azido-functionalized polystyrene was prepared following two steps: in the first step, polystyrene (10.00 g, 2.22 mmol, *M*_n_ = 4500 g mol^−1^) was dissolved in dried CH_2_Cl_2_ (100 mL) in a two necked round bottom flamed dried flask equipped with a magnetic stirrer, followed by the addition of trimethylamine (9.3 mL, 66.7 mmol). The temperature of the reaction was reduced to 0 °C by putting the flask in an ice bath. Finally, *p*-toluenesulfonyl chloride (4.24 g, 22.2 mmol) was added in small portions under argon flow. The temperature of the system was allowed to raise slowly to room temperature and the reaction mixture was allowed to react overnight under stirring. The reaction mixture was diluted by the addition of CH_2_Cl_2_ (100 mL) and transferred into a separating funnel (500 mL) where residual salts were removed by extraction with water (3 × 100 mL). The organic layer was then dried using MgSO_4_ and the solvent was removed by rotary evaporator. The polymer was redissolved in an appropriate amount of THF and precipitated twice in methanol (500 mL). The white precipitate of tosyl terminated polystyrene (PS-OTs) was filtered using a sintered glass funnel under vacuum and dried in a vacuum oven at 40 °C overnight. There was 9.3 g of solid product, with a 90% yield. The sample was analyzed by ^1^H NMR (Figure S2). In the second step, *ω*-tosyl polystyrene (9 g, 1.94 mmol) prepared in the above step was charged in a two necked round bottom flask containing DMF (60 mL) and equipped with a magnetic stirrer. NaN_3_ (2.50 g, 38.7 mmol) was then added under stirring and the reaction mixture was placed in a preheated oil bath at 60 °C overnight. The system was then allowed to be cooled down to room temperature, diluted with CH_2_Cl_2_ (200 mL) and transferred into a separating funnel where it was washed repeatedly with water to remove the residual tosylate salt, excess of NaN_3_ and DMF. The organic layer was then dried by adding anhydrous MgSO_4_. CH_2_Cl_2_ was removed by evaporation using a rotary evaporator. The polymer was redissolved in an appropriate amount of THF and precipitated twice in methanol (500 mL). The white precipitate of azido terminated polystyrene was filtered using a sintered glass funnel under vacuum and dried in a vacuum oven at 40 °C overnight. There was 8.0 g of solid product, with an ~89% yield. The sample was characterized by ^1^H NMR and SEC (Figure S3).

#### 2.2.3. Synthesis of Polystyrene-Block-Maltoheptaose (PS-b-MH) Block Copolymer

Polystyrene-*b*-maltoheptaose was prepared by click chemistry of azido functionalized PS and alkynyl functionalized maltoheptaose. A round bottom, one necked flask equipped with rotoflow and a magnetic stirrer was charged with *ω*-azido polystyrene (1 eq, 6.0 g, ~1.33 mmol), propargyl-maltoheptaose (1.2 eq, 2.0 g, 1.6 mmol), and DMF (40 mL) and degassed by three freeze-pump-thaw cycles and then copper nanopowder (2 eq vs. acetylene group, 205 mg, 3.20 mmol) was added to the solution under argon flow and subjected to another freeze–pump–thaw cycle. The solution was stirred under argon atmosphere at 65 °C for 3 days. At the end of the reaction, the crude heterogeneous solution was diluted with THF and filtered through diatomaceous earth. The obtained filtrate was stirred with 5.0 g cuprisorb resin at 40 °C overnight. The solution was filtered to remove the cuprisorb resin and the solvent was removed by distillation using a roto-evaporator. The crude product was redissolved in an appropriate amount of water and precipitated in methanol to remove the excess maltoheptaose. The unreacted polystyrene was removed by precipitation of the copolymer in a cyclohexane/heptane (60/40, *v/v*) mixture. The resulting white solid (6.5 g solid product, with an ~85% yield) was dried in vacuum at 40 °C overnight and characterized by ^1^H NMR and SEC (Figure S4).

#### 2.2.4. Synthesis of Polystyrene-Block-β-Cyclodextrin (PS-b-βCD) Block Copolymer

The above method was used to synthesize PS-*b*-βCD by click chemistry of azido functionalized PS (1 eq, 4 g, 0.89 mmol) with mono-6-N-propargylamino-6-deoxy-β-cyclodextrin (1.2 eq, 1.25 g, 1.07 mmol) in the presence of copper nanopowder (2 eq vs. alkynyl group, 136 mg, 2.13 mmol) in DMF (30 mL) at 65 °C for 72 h. There was 4.5 g of white solid, with an ~85% yield. The resulting copolymer was characterized by SEC and ^1^H NMR (Figure S5).

### 2.3. Preparation of Glyconanoparticles (GNPs) by Nanoprecipitation

The 4 GNPs (GNP_PSCD_, GNP_PSMH_, GNP_PSCD50_ and GNP_PSCD10_) were obtained by the dissolution of, respectively, 30 mg of copolymer PS-*b*-CD, 30 mg PS-*b*-MH, 15 mg PS-*b*-βCD with 15 mg PS-*b*-MH and finally 3 mg of PS-*b*-βCD with 27 mg PS-*b*-MH in 4 mL THF/H_2_O solution (80: 20) *w/w* stirred at 1000 rpm for 24 h. This resulting solution was added drop by drop using a syringe pump debit at 10.2 mL h^−1^ in 160 mL of Milli-Q water under stirring at 500 rpm and then stirred for 2 h at room temperature. After THF removal under reduced pressure at 40 °C, the glyconanoparticles suspension was obtained with a total concentration of 0.1875 mg L^−1^ and kept at 4 °C without further purification.

### 2.4. Functionalization of GNPs with Sodium Anthraquinone-2-Sulfonate (AQS)

An amount of 3 mg of sodium anthraquinone-2-sulfonate (AQS) was added in 10 mL of each GNPs suspension. An ultrasound exposure was then performed for 2 h and the resulting solutions were then stirred for 12 h at room temperature. The AQS excess was eliminated using a dialysis against purified water for 72 h at room temperature (cut-off = 3.5–5 kDa, 72 h; 20 × 2 L of water, 300 rpm). The 72 h duration of dialysis is sufficient to remove AQS non-involved in interaction with GNPs, as the solution obtained after dialysis carried out with AQS in the same experimental conditions shows no redox signal characteristic of AQS on cyclic voltammetry (CV). These results attest that after 12 h of dialysis, AQS concentration is too low to be detected by CV, proving, therefore, the dialysis efficiency to remove AQS. The different GNPs/AQS suspensions were kept at 4 °C without further purification. The solutions were sonicated for 5 min before each use.

### 2.5. Characterization by ^1^H NMR and by Size Exclusion Chromatography

^1^H NMR spectra of polymer samples were recorded on a Bruker Avance 400 MHz spectrometer (Billerica, MA, USA) with a frequency of 400.13 MHz and calibrated with the signal of deuterated solvent. The size exclusion chromatography (SEC) was performed at 40 °C using an Agilent 390 MDS system (290 LC pump injector, ProStar 510column oven, 390 MDS refractive index detector) (Santa Clara, CA, USA) equipped with a Knauer Smartline UV detector 2500 (Berlin, Germany) and two Agilent Poly Pore PL1113−6500 columns (linear, 7.5 × 300 mm; particle size, 5 μm; exclusion limit, 200−2,000,000) in DMF containing lithium chloride (0.01 mol L^−1^) at the flow rate of 1.0 mL min^−1^.

### 2.6. Characterization of the Glyconanoparticles by Scanning Electron Microscopy (SEM) and by Transmission Electron Microscopy (TEM)

SEM images of GNPs were collected using an ULTRA 55 FESEM based on the GEMENI FESEM column with beam booster (Nanotechnology Systems Division, Carl Zeiss NTS GmbH, Oberkochen, Germany,) with a tungsten gun. A drop of GNP solution was spread onto the carbon substrate (diameter = 0.5 mm) and dried at room temperature before imaging. TEM pictures of GNPs were performed at 200 kV using a Phillips CM200 microscope equipped with a TEM-CAM 216 (TVIPS, Gauting, Germany) camera and at 200 kV using a JEOL 2100 Plus microscope (Tokyo, Japan) equipped with a RIO16 (GATAN, Pleasanton, CA, USA) camera. For measurements, a 5 µL drop of GNPs solution was deposited onto plasma treated carbon-coated copper microgrid. An amount of 5 μL of 2 *w/v*% UranyLess (Delta Microscopies, Ayguesvives, France) lanthanide-based negative stain was applied for a few minutes and dried at room temperature. The excess of liquid was removed by capillarity using blotting paper.

For both SEM and TEM microscopies, GNPs’ diameters were measured using ImageJ software from multiple images and multiple places on the substrate, allowing the realization of histograms depicting the size distribution of each glyconanoparticle. The obtained histograms were fitted by a log-normal model. The correlation coefficient value for each distribution allowed for the validation of the chosen model.

### 2.7. Characterization of the Glyconanoparticles by Dynamic Light Scattering (DLS) and Nanoparticles Tracking Analysis (NTA)

Dynamic light scattering (DLS) and nanoparticles tracking analysis (NTA) were used to evaluate the GNPs distribution. Light scattering experiments were carried out using an ALV/CGS-8FS/N069 goniometer, which consists of an ALV/LSE-5004 multiple-τ digital corrector with a 125 ns initial sampling time and 35 mW HeNe linearly polarized laser operating at a wavelength of 632.8 nm. Nanoparticle suspensions were directly poured into the quartz cells thermostated at 25 ± 0.1 °C. In DLS, relaxation time distributions were determined using the CONTIN analysis of the autocorrelation functions and hydrodynamic diameters (D_h,DLS_), and calculated using the Stokes–Einstein equation [3,8]. NTA was carried out using a Nanosight LM10HS optical microscope setup equipped with a blue–purple laser (λ_ex_ = 405 nm), a camera, and a chamber mounted on a modified microscope stage (Nanosight, Amesbury, U.K.). The original suspensions of nanoparticles were diluted with Milli-Q water ((copolymer) = 0.02 mg mL^−1^) and introduced into the chamber with a syringe. Video clips of the nanoparticles subjected to their natural Brownian motion were captured over 60 s at 25.0 °C and analyzed using analytical software version 2.1, giving access to the number-weighted size distributions.

### 2.8. Electrochemistry Measurements

Electrochemical experiments were carried out using a conventional three-electrode potentiostat coupled to a PGSTAT 100 operated by Nova 2.1.4 software. The working electrodes were a vitreous carbon electrode (diameter 3 mm) thoroughly cleaned using 20 µm diamond paste, and rinsed several times successively with acetone, ethanol, and distilled water. The counter electrode was a platinum wire, and the reference electrode was a saturated calomel electrode (SCE). All experiments were performed at room temperature under controlled argon atmosphere.

## 3. Results and Discussions

### 3.1. Self-Assembly of Block-Copolymers

**Figure 1:**
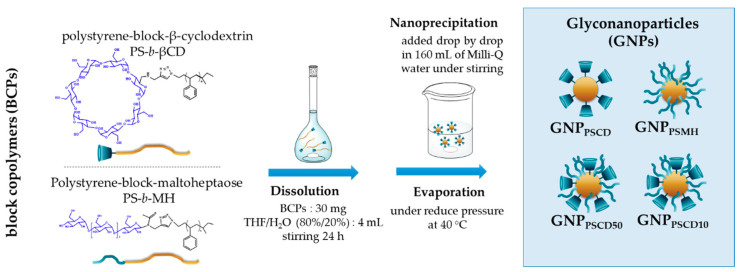
Schematic illustration of the self-assembly procedure to form pure (GNP_PSCD_, GNP_PSMH_) and hybrid glyconanoparticles (GNP_PSCD50_, GNP_PSCD10_) in aqueous media.

The different nanoparticles were obtained by self-assembled block copolymer systems in aqueous solution via nanoprecipitation, according to a method adaptation of the one previously described [1,8,12,13,14]. It consists in a first step in the dissolution of 7.5 mg of the corresponding copolymer in a mixture of 80:20 THF/H_2_O ratio, allowing for the good solubilization of both hydrophobic and hydrophilic blocks of PS-*b*-βCD and PS-*b*-MH glycopolymers, as confirmed by low scattering intensities measured by static light scattering (SLS) experiments (Figure S6) (I = 17 ± 7 kHz and 10 ± 3 kHz, respectively for of PS-*b*-βCD and PS-*b*-MH for a concentration of 7.5 mg mL^−1^). After 24 h of agitation, the resulting solution was added in a large volume of water of 160 mL under uniform mechanical stirring and after evaporation of tetrahydrofuran, stable suspensions of GNPs were obtained with a polystyrene heart covered by sugars (Figure 1).

### 3.2. Morphology and Size Determination of GNPs by SEM and TEM

**Figure 2:**
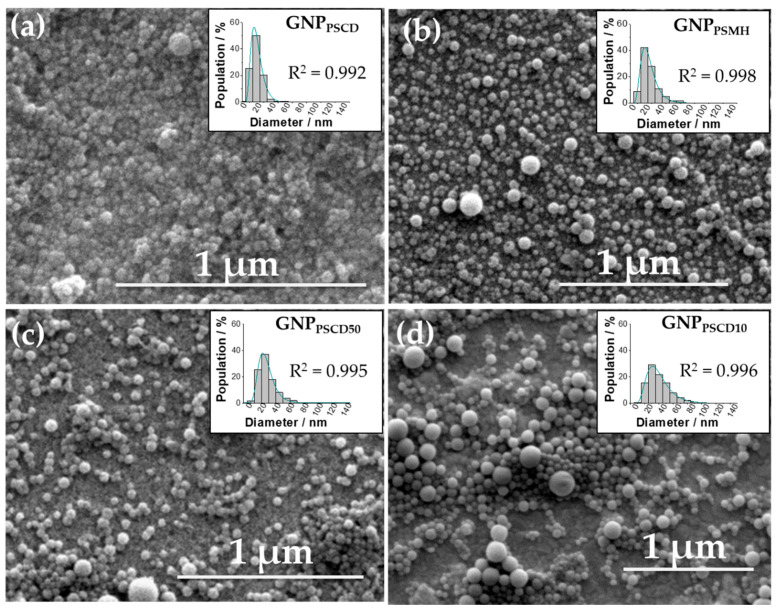
SEM images of the self-assembled nanoparticles (**a**) GNP_PSCD_ (**b**) GNP_PSMH_ (**c**) GNP_PSCD50_ and (**d**) GNP_PSCD10_ with respective particle size distributions fitted by a log-normal model.

In order to confirm the spontaneous formation of nanoparticles by self-assembly, the nanoprecipitation products have been analyzed by SEM for pure glyconanoparticles GNP_PSCD_ (Figure 2a), GNP_PSCDMH_ (Figure 2b) and for hybrid particles GNP_PSCD50_, GNP_PSCD10_ (Figure 2c,d, respectively). As can be seen in Figure 2a,b, spherical nanoparticles were obtained for GNP_PSCD_ and GNP_PSCDMH_, as reported in previous works for these kind of nanoparticles [4,5,8]. In addition, for new hybrid nanoparticles, the same morphology is observed. By fitting the distributions curves by a log-normal model (R^2^ > 0.99), the largest distributions in number for GNP_PSCD_, GNP_PSMH_ GNP_PSCD50_ and GNP_PSCD10_ are obtained for respective diameters of 20, 24, 30 and 37 nm. In addition, particles obtained with single PS-*b*-βCD and PS-*b*-MH copolymer have less extended size distributions than hybrid particles. A total of 95% of GNP_PSCD_ and GNP_PSMH_ have a diameter indeed lower than 40 nm and 60 nm, respectively, while for hybrid particles, distributions extend up to 70 nm and 80 nm for GNP_PSCD50_ and GNP_PSCD10_, respectively.

**Figure 3:**
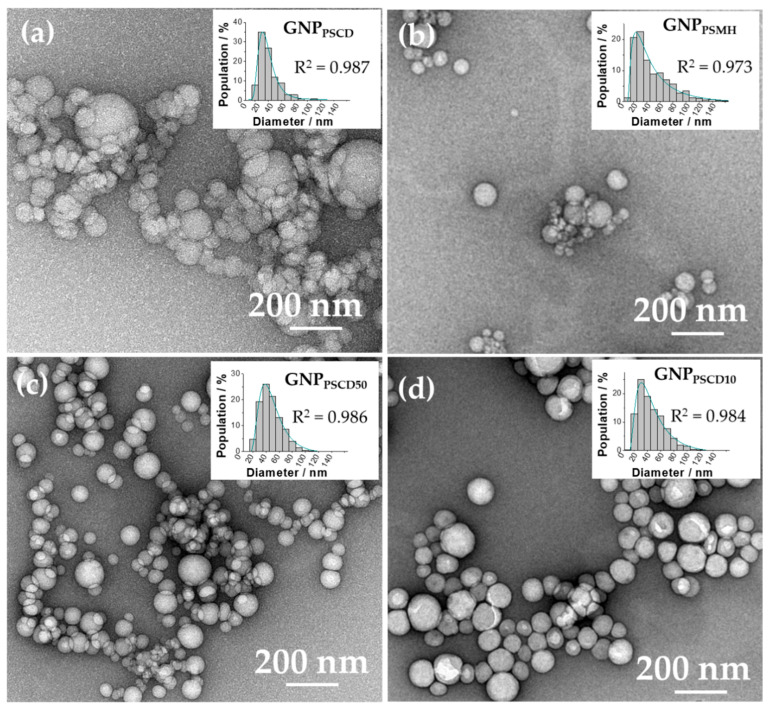
TEM images of the self-assembled nanoparticles (**a**) GNP_PSCD_ (**b**) GNP_PSMH_ (**c**) GNP_PSCD50_ and (**d**) GNP_PSCD10_ in association with the particle size distributions.

The spherical morphologies of the different GNPs were also confirmed by TEM measurements (Figure 3). The respective images of GNP_PSCD_ (Figure 3a), GNP_PSMH_ (Figure 3b) GNP_PSCD50_ (Figure 3c) and GNP_PSCD10_ (Figure 3d) in association with their particle size distribution fitted by the log-normal model reveal respective diameters of 26, 32, 36 and 34 nm for GNP_PSCD_, GNP_PSMH_, GNP_PSCD50_ and GNP_PSCD10_. The diameters measured are close to those obtained by SEM, except for GNP_PSMH_ (32 nm instead of 24 nm); this can be attributed to the precision of the adjustment model (R^2^ = 0.973 against 0.993). Regarding the dispersions, 95% of the particles have diameters below 100 nm for GNP_PSCD_, GNP_PSMH_, GNP_PSCD50_ and GNP_PSCD10_. These results are in good agreement with the previous measurements, confirming that the majority size distribution is composed of nanoparticles with diameters between 20 and 40 nm. It should be noted that the concentration of block copolymer is certainly affecting the size of the nanoparticles. Size increases with concentration, and shape can change from spherical to long cylinders into vesicles.

### 3.3. Hydrodynamic Diameter Determination of GNPs by Dynamic Light Scattering (DLS)

**Figure 4:**
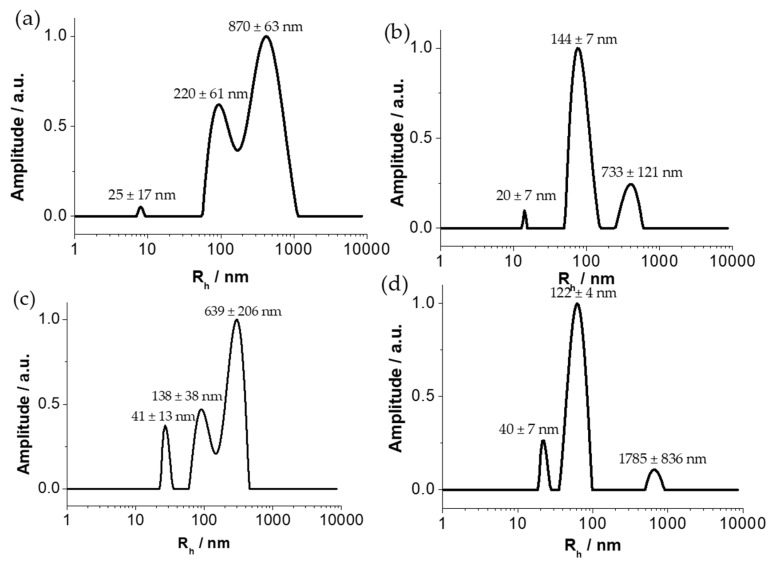
Size distribution function obtained by DLS for (**a**) GNP_PSCD_ (**b**) GNP_PSMH_ (**c**) GNP_PSCD50_ and (**d**) GNP_PSCD10_. A total of 4 measurements were carried out for the determination of the average hydrodynamic diameter indicated in the figure.

The sizes of the different glyconanoparticles suspensions GNP_PSCD_, GNP_PSMH_, GNP_PSCD50_ and GNP_PSCD10_ were determined in aqueous solution by dynamic light scattering (DLS) at a fixed angle of 90°, although the same results were obtained at different angles. Figure 4 represents the intensity of the signal as a function of the hydrodynamic radius. The presence of three peaks for each suspension of GNPs reveals a size distribution in three groups for each type of nanoparticles. The first distributions exhibit a hydrodynamic diameter (D_h,DLS_), of 25 ± 17 nm, 20 ± 7 nm, 41 ± 13 nm and 40 ± 7 nm whereas the second and third distributions have diameters of 220 ± 61 nm, 144 ± 7, 139 ± 38 and 122 ± 4 nm and 870 ± 63 nm, 733 ± 121 nm, 639 ± 206 nm and 17,885 ± 836 nm for GNP_PSCD_, GNP_PSMH_, GNP_PSCD50_, GNP_PSCD10_, respectively. The distributions with the largest diameters suggest the formation of large agglomerates, as the solutions obtained were not filtered. It should be noted that the distribution plot corresponds to a mass-weighted distribution and thus implies that the distribution with the largest diameter is in fact the minority distribution. These results were confirmed by nanoparticle tracking analysis (NTA) revealing (Figure S7) that above a diameter of 250 nm, whatever the nanoparticles suspension observed, no glyconanoparticle is detected, proving that the third distribution observed in DLS is a negligible number. In addition, these results corroborate those obtained for SEM and TEM measurements, where size distributions for GNPs with a diameter higher than 120 nm are not detectable in histograms. It should be noted that dispersions of spherical glyconanoparticles in aqueous media remain stable for at least 3 months.

### 3.4. Characterization of the Host–Guest Properties of GNPs by Cyclic Voltammetry

**Figure 5:**
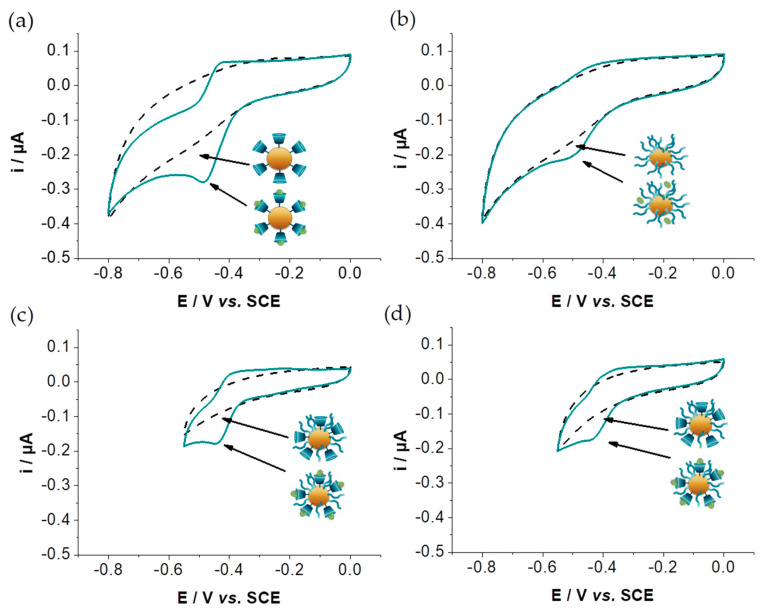
Cyclic voltammograms recorded at a glassy carbon electrode (3 mm diameter disc) of (**a**) GNP_PSCD_, (**b**) GNP_PSMH_, (**c**) GNP_PSCD50_ (**d**) GNP_PSCD10_ aqueous solution under Ar, before (dashed line) and after AQS incubation. Scan rate: 10 mV s^−1^; pH 6.0. Total polymer concentration before dialysis = 0.1875 mg mL^−1^.

The various designed glyconanoparticles functionalized by AQS, GNP_PSCD_/AQS (Figure 5a) GNP_PSMH_/AQS (Figure 5b), GNP_PSCD50_/AQS (Figure 5c), GNP_PSCD10_/AQS (Figure 5d) were studied by cyclic voltammetry in an aqueous 0.1 mol L^−1^ KCl solution at pH 6. Taking into account that the electrochemical behavior of AQS in aqueous solution exhibits a reversible peak system at −0.422 V vs. SCE attributed to the two-electron reduction of AQS (Figure S8) [15], cyclic voltammograms were recorded in the potential window 0.0 V to −0.80 V for GNP_PSCD_/AQS and GNP_PSMH_/AQS and 0.0 V to −0.60 V for the hybrid GNP_PSCD50_/AQS and GNP_PSCD10_/AQS. As expected, glyconanoparticles which were not incubated with AQS, show no electrochemical activity in this potential range (Figure 5). In contrast, cyclic voltammograms for all glyconanoparticles incubated with AQS exhibit in the same potential range a similar reversible peak system. This redox couple was attributed to the reversible reduction of AQS, reflecting its efficient capture by glyconanoparticles. The orientation of the glycan is always at the shell (outer layer) of the glyconanoparticles and therefore AQS should form a shell by inclusion in cyclodextrin sites. Surprisingly, GNP_PSMH_/AQS without cyclodextrin also presents a redox couple which can result from the insertion of AQS within the stacked maltoheptaose blocks and its stabilization by hydrogen bond. However, a slight negative shift of the E_1/2_ value of this system (−0.460 V) is recorded compared to that observed for free AQS in solution (−0.422 V). On the other hand, it should be noted that the inclusion of AQS within the hydrophobic βCD cavity does not alter its electroactivity, the E_1/2_ values of the redox couple being almost identical to that observed for free AQS (Table 1). This illustrates the ease of functionalization of glyconanoparticles based on the βCD block and the preservation of the electrochemical behavior of redox compounds loaded in βCD.

With the aim to compare the immobilization capacity of the different glyconanoparticles, the AQS loading was estimated by comparing the current intensity of the cathode peak, assuming that the four types of nanoparticle have a similar diffusion coefficient. As expected, the highest current value was measured with GNP_PSCD_/AQS, which exhibits a pure βCD shell. Table 1 summarizes the current values in percentage with respect to the current for GNP_PSCD_/AQS.

It appears that GNP_PSMH_ containing no cyclodextrin, can even immobilize about 30% of the amount fixed by GNP_PSCD_. Regarding the hybrid nanoparticles, GNP_PSCD50_ and GNP_PSCD10_, present percentages of 67 ± 16% and 32 ± 12%, respectively, which correspond relatively well to the percentage of PS-*b*-βCD within the GNP shell (50 and 10%, respectively) increased by a contribution due to non-specific interactions.

**Figure 6:**
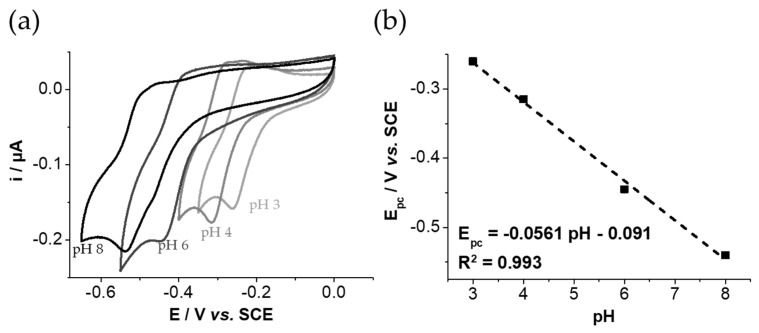
Electrochemical behavior of GNP_PSCD_ solution (0.14 mg mL^−1^ in water) under Ar: (**a**) cyclic voltammograms recorded at a glassy carbon electrode (3 mm diameter disc) at different pH 3, 4, 6 and 8 and (**b**) plot of cathodic peak potential value cathodic potential vs pH. Scan rate: 10 mV s^−1^.

Moreover, the electrochemical behavior of GNP_PSCD_ containing AQS was studied at different pH ranging from 3.0 to 8.0. Figure 6 shows the effect of pH on the redox signal of GNP_PSCD_/AQS. The increase in the pH value induces a shift of the reduction potential to more negative values. As previously reported for anthraquinone sulfonate electrochemical behavior and its derivative [15] in aqueous solutions, the signal corresponds to the 2 *H*^+^/2 *e*^−^ reduction mechanism (Equation (1)) where *AQS* and *AQSH*_2_ are the oxidized and reduced forms, respectively. The linear slope of-56 mV per pH unit (Figure 6b) indicates clearly that the electrochemical process indeed involves one proton per electron transfer [15,16,17]. This demonstrates that the electrochemical behavior of *AQS* is not affected by its inclusion within the βCD cavity.

(1) $$AQS+2\;e^{-}+2\;H^{+}\to AQSH_{2}$$

## 4. Conclusions

This work was devoted to the self-assembly and characterization by various physicochemical methods of new hybrid glyconanoparticles based on mixtures of PS-*b-*βCD and PS-*b*-MH and to their comparison with glyconanoparticles composed of only one type of copolymer. All resulting GNPs are spherical and have average diameters in the range of 20 to 40 nm, with hybrid GNPs exhibiting a larger diameter than the GNPs obtained from the self-assembly of single block copolymer system. The ability of these GNPs to interact with a redox probe, such as anthraquinone sulfonate chosen as a model, was examined by cyclic voltammetry. It appears that it is possible to modulate the site density of βCD at the surface of the shell of the hybrid glyconanoparticles while maintaining its inclusion properties. This approach allowed one to modulate the electroactivity of the GNPs, as illustrated with a water-soluble redox compound such as AQS.

This elegant, innovative and versatile concept will be extended in the near future to the anchoring of biotinylated proteins such as enzymes or antibodies via biotin/βCD interactions. The modulation of the inclusion sites should make this concept possible to generate a shell of biological macromolecules based on a single interaction per biomolecule and thus without blocking their flexibility or their active site.

The formation of hybrid glyconanoparticles modified by a protein shell could lead to numerous applications in biotechnology, cosmetics and in the biomedical field. The combination of redox nanoparticles and enzyme glyconanoparticles could be used, for example, for the development of enzymatic biofuel cells for the conversion of chemical energy into electrical energy. Moreover, glyconanoparticles modified by antigens or antibodies could also be used in the transduction step of biosensors or even in their elaboration via the anchoring of these nanoparticles on a surface by host–guest interactions.

## Figures and Tables

**Table 1:** Values of cathodic current reported as a percentage with respect to GNP_PSCD_ (current normalized to 100) and values of *E*_1/2_ potentials. *E*_1/2_ = (*E*_pa_ + *E*_pc_)/2 where *E*_pa_ and *E*_pc_ are the anodic and cathodic peak potentials. Measurements performed in triplicate.

Nanoparticles	GNP_PSCD_/AQS	GNP_PSMH_/AQS	GNP_PSCD50_/AQS	GNP_PSCD10_/AQS
Current (%)	100 ± 7	30 ± 5	67 ± 16	32 ± 12
*E*_1/2_ (V vs. SCE)	−0.426	−0.460	−0.419	−0.414

## Supplementary Materials

The following are available online at https://www.mdpi.com/article/10.3390/nano11051162/s1, Figure S1. ^1^H NMR of (a) PS-OH, (b) PS-OTs and (c) PS-N3 in CDCl_3_ at 25 °C (400 MHz), Figure S2. ^1^H NMR of PS-*b*-MH in DMF-d7 at 25 °C (400 MHz), Figure S3. SEC traces of PS_4.5k_-N_3_ (Blue), MH_1.2k_ (Red) and PS_4.5k_-*b*-MH_1.2k_ (green) using DMF as an eluent and PS calibration at 40 °C, Figure S4. ^1^H NMR of PS-*b*-βCD in DMF-*d_7_* at 25 °C (400 MHz), Figure S5. SEC traces of PS_4.5k_-N_3_ (Blue), and PS_4.5k_-*b*-βCD_1.2k_ (green) using DMF as an eluent and PS calibration at 40 °C, Figure S6. Scattering intensity as a function of mass concentration of PS-*b*-βCD and PS-*b*-MH glycopolymers in a THF/H_2_O solution mixture (80: 20 *w/w*%), Figure S7. Size distribution of the solutions of (a) GNP_PSCD_ (b) GNP_PSMH_ (c) GNP_PSCD50_ and (d) GNP_PSCD10_ determined by NTA analysis, Figure S8. Cyclic voltammetry performed at 10 mV s^−1^ with glass carbon in aqueous solution (KCl 0.1 mol L^−1^) with AQS (0.3 mg mL^−1^). pH was adjusted to 6.0.
