# Supplementary material for: Functionalizable Glyconanoparticles for a Versatile Redox Platform
Source: Nanomaterials (Basel). 2021 Apr 29;11(5):1162. doi: 10.3390/nano11051162 (PMC8146528; doi:10.3390/nano11051162)
Supplement: Supplementary file 1 [file nanomaterials-11-01162-s001.zip › nanomaterials-1197436-supplementary.pdf]

nanomaterials

Supplementary Information

Functionalizable Glyconanoparticles for a Versatile Redox Platform

Marie Carrière [1,2], Paulo Henrique M. Buzzetti [1], Karine Gorgy [1], Muhammad Mumtaz [2], Christophe Travelet [2], Redouane Borsali [2,*] and Serge Cosnier [1,*]

[1] UMR 5250, Département de Chimie Moléculaire, CNRS, Université Grenoble Alpes, CEDEX 09, 38058 Grenoble, France; marie.carriere@univ-grenoble-alpes.fr (M.C.); paulo-henrique.maciel-buzzetti@univ-grenoble-alpes.fr (P.H.M.B.); karine.gorgy@univ-grenoble-alpes.fr (K.G.)

[2] CERMAV, UPR 5301, CNRS, Université Grenoble Alpes, CEDEX 09, 38058 Grenoble, France; mumtaz@cermav.cnrs.fr (M.M.); christophe.travelet@cermav.cnrs.fr (C.T.)

* Correspondence: Redouane.borsali@cermav.cnrs.fr (R.B.); Serge.Cosnier@univ-grenoble-alpes.fr (S.C.)

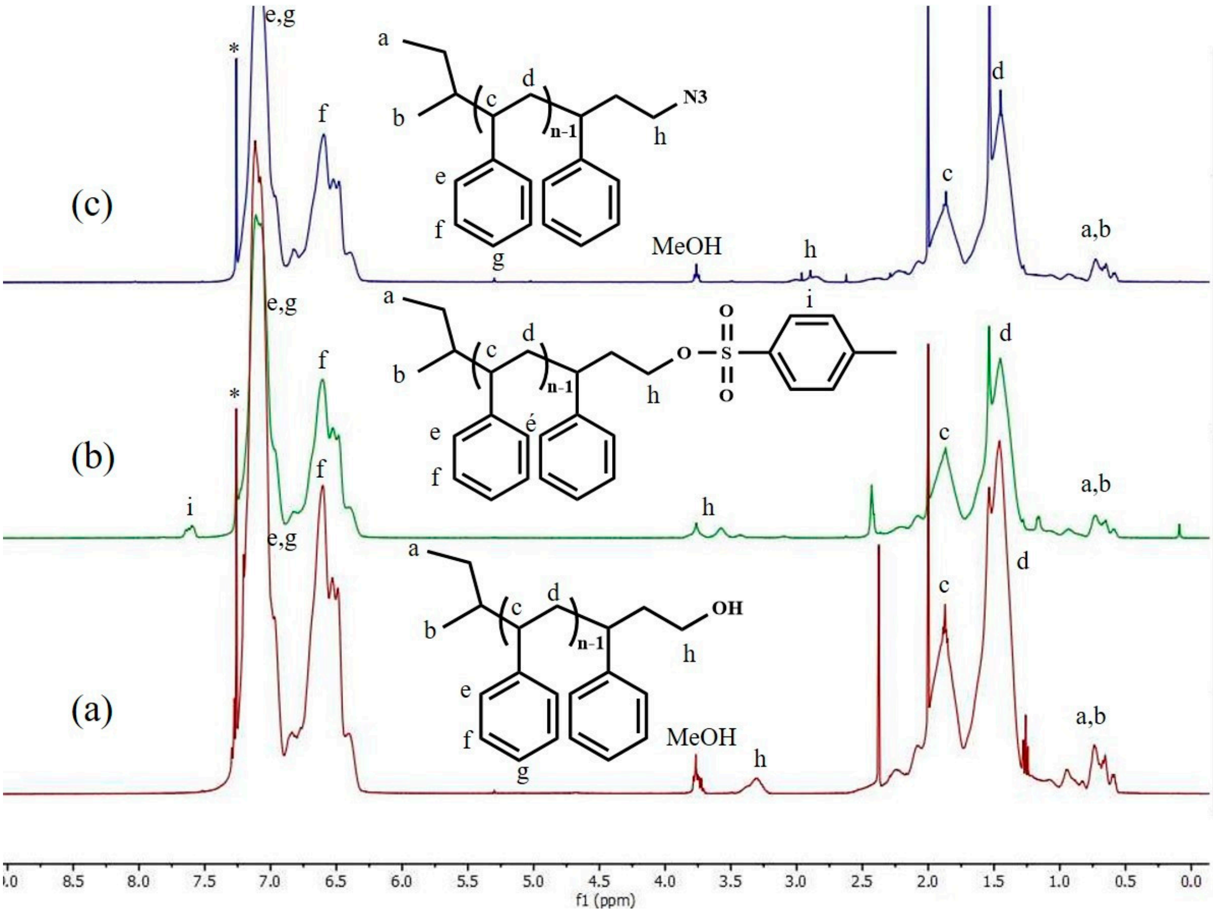

Figure S1. ^{1}H NMR of (a) PS-OH, (b) PS-OTs and (c) PS-N3 in $CDCl_3$ at 25°C (400 MHz)

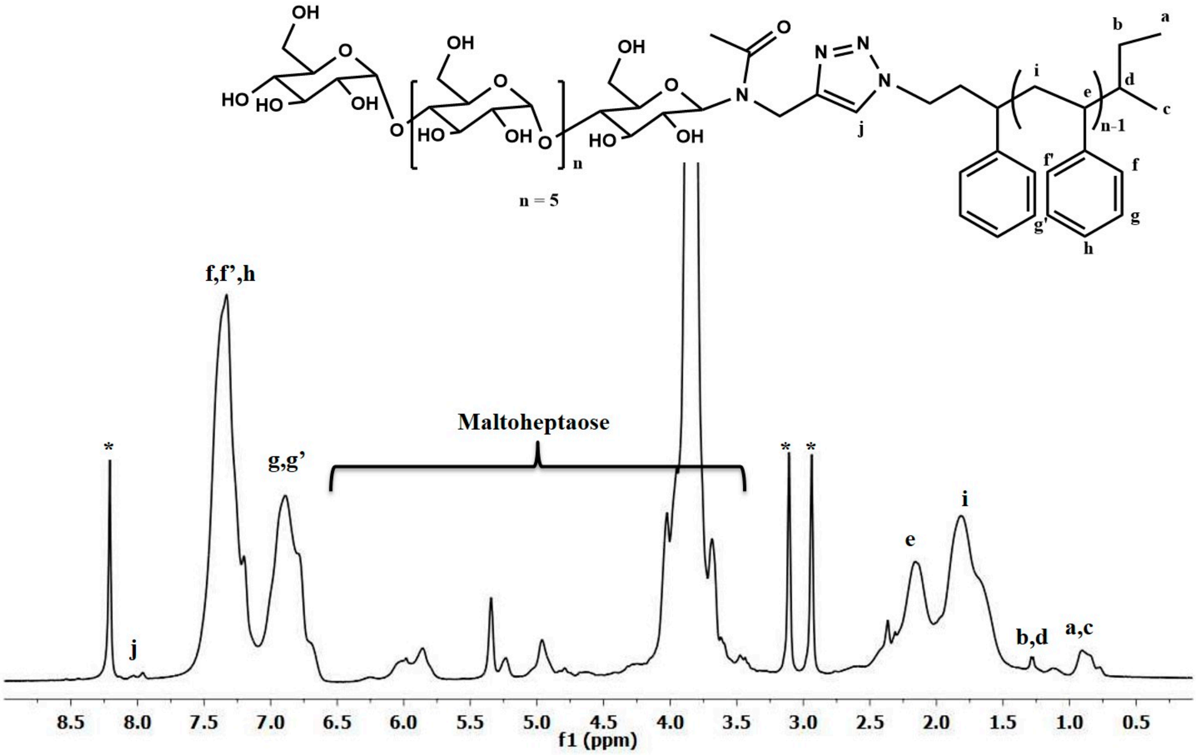

Figure S2. ^{1}H NMR of PS-*b*-MH in DMF-d7 at 25°C (400 MHz)

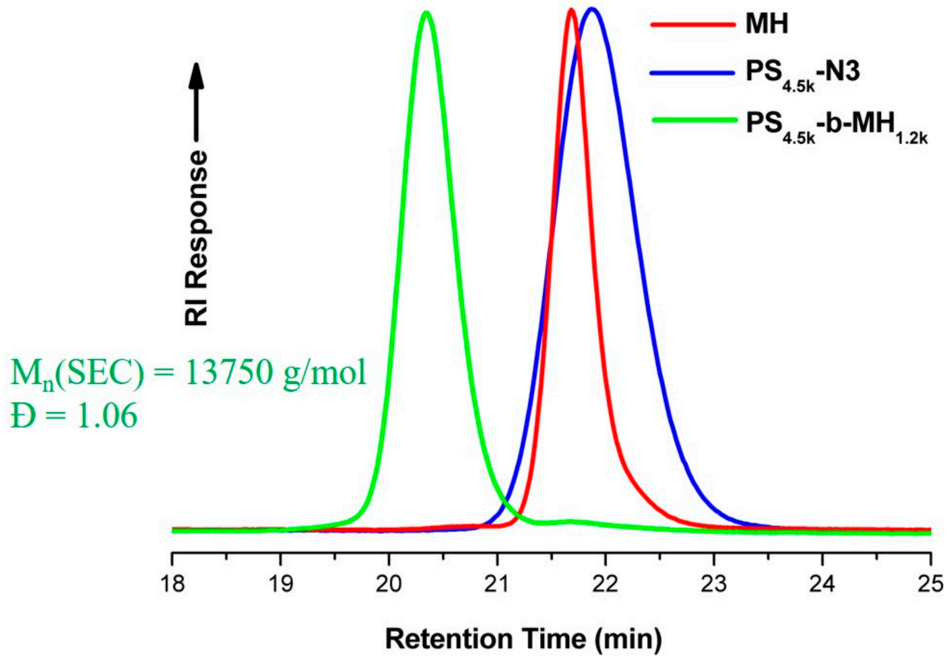

Figure S3. SEC traces of $PS_{4.5k}$-N_3 (Blue), $MH_{1.2k}$ (Red) and $PS_{4.5k}$-*b*-$MH_{1.2k}$ (green) using DMF as an eluent and PS calibration at 40°C.

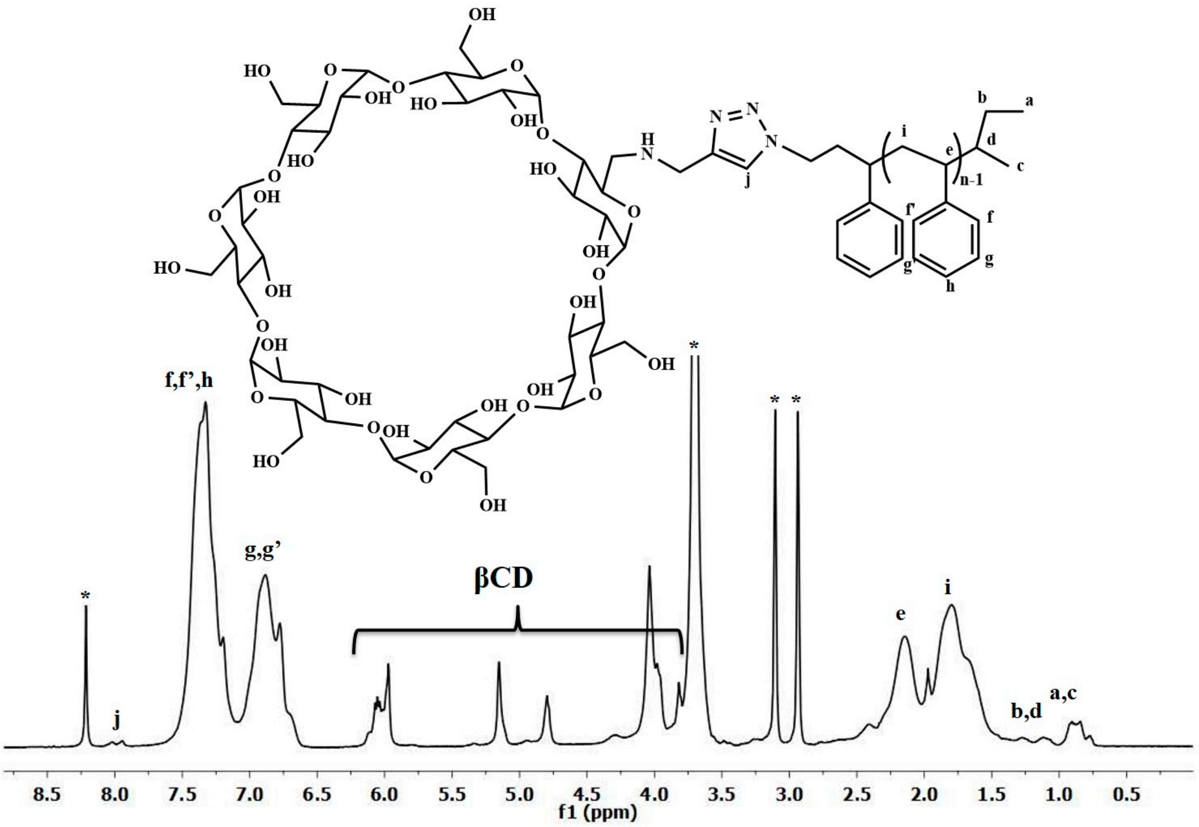

Figure S4. ^{1}H NMR of PS-*b*-βCD in DMF-d_7 at 25°C (400 MHz)

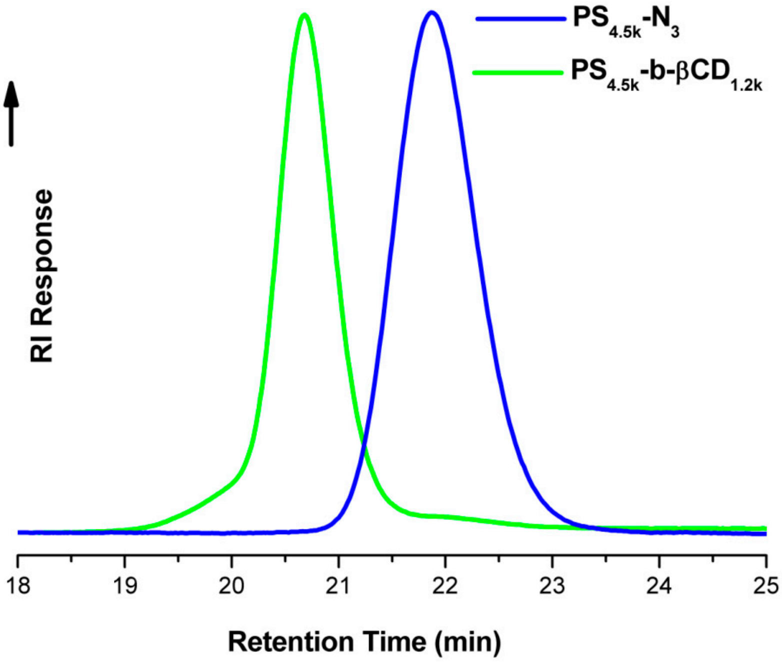

Figure S5. SEC traces of $PS_{4.5k}$-N_3 (Blue), and $PS_{4.5k}$-*b*-$\beta CD_{1.2k}$ (green) using DMF as an eluent and PS calibration at 40°C.

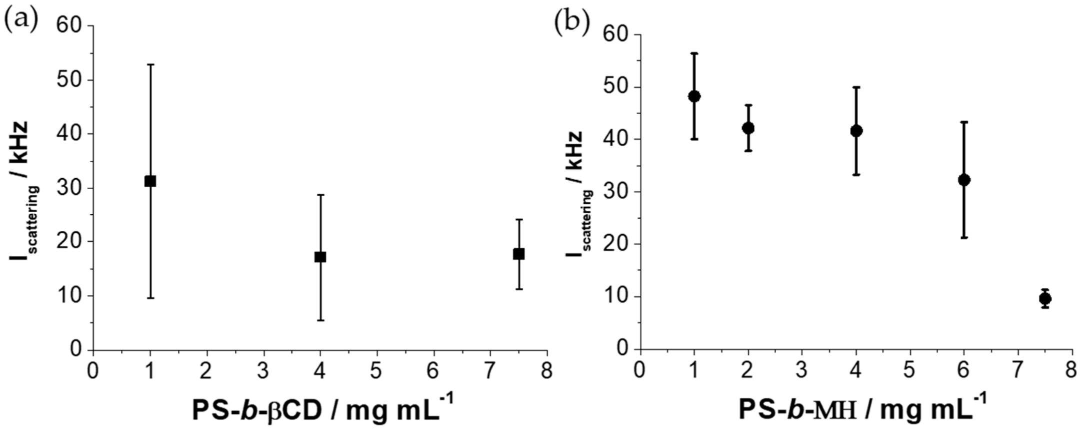

Figure S6. Scattering intensity as a function of mass concentration of PS-*b*-βCD and PS-*b*-MH glycopolymers in a THF/H_2O solution mixture (80:20 w/w %).

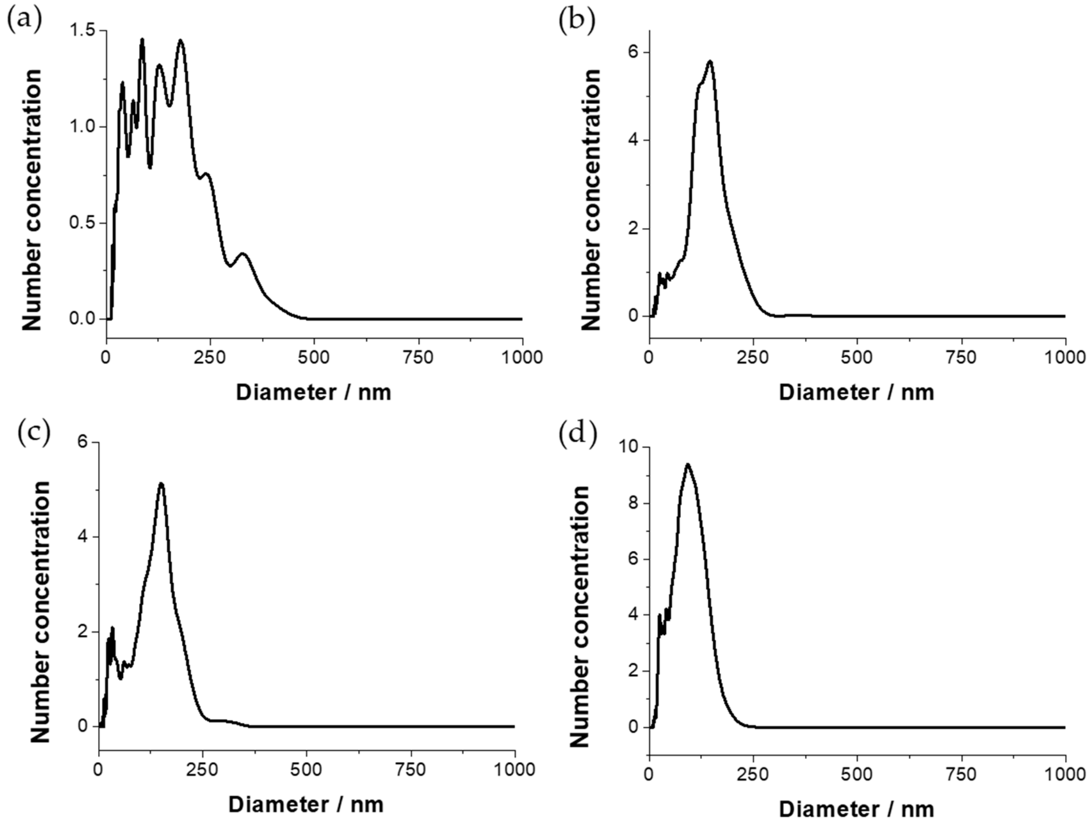

Figure S7. Size distribution of the solutions of (a) GNP_{PSCD} (b) GNP_{PSMH} (c) GNP_{PSCD50} and (d) GNP_{PSCD10} determined by NTA analysis.

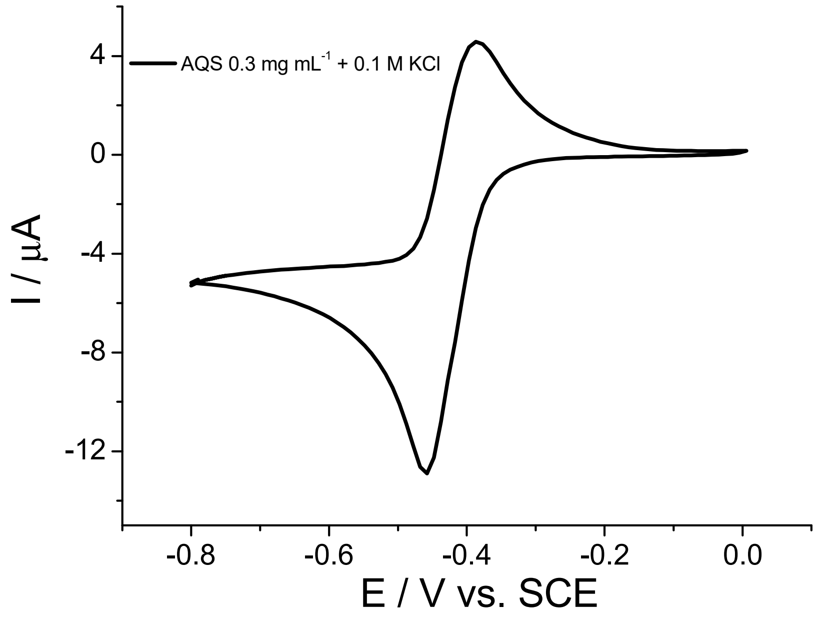

Figure S8. Cyclic voltammetry performed at 10 mV s^{-1} with glass carbon in aqueous solution (KCl 0.1 mol L^{-1}) with AQS (0.3 mg mL^{-1}). pH was adjusted to 6.0.
